# Socio-cultural inhibitors to use of modern contraceptive techniques in rural Uganda: a qualitative study

**DOI:** 10.11604/pamj.2016.25.78.6613

**Published:** 2016-10-17

**Authors:** Allen Kabagenyi, Alice Reid, James Ntozi, Lynn Atuyambe

**Affiliations:** 1 Department Population Studies, School of Statistics and Planning, College of Business and Management Sciences, Makerere University, P.O. Box 7062, Kampala, Uganda; 2 Center for Population and Applied Statistics, College of Business and Management Sciences, Makerere University, P.O. Box 7062, Kampala, Kampala, Uganda; 3 Department of Geography, University of Cambridge, Downing Pl, Cambridge CB2 3EN, United Kingdom; 4 Department of Community and Behavioral Sciences, School of Public Health, College of Health Sciences, Makerere University, P. O Box 7062, Kampala, Kampala, Uganda

**Keywords:** Uganda, contraceptive use, cultural inhibitions, beliefs, practices and family planning

## Abstract

**Introduction:**

Family planning is one of the cost-effective strategies in reducing maternal and child morbidity and mortality rates. Yet in Uganda, the contraceptive prevalence rate is only 30% among married women in conjunction with a persistently high fertility rate of 6.2 children per woman. These demographic indicators have contributed to a high population growth rate of over 3.2% annually. This study examines the role of socio-cultural inhibitions in the use of modern contraceptives in rural Uganda.

**Methods:**

This was a qualitative study conducted in 2012 among men aged 15-64 and women aged 15-49 in the districts of Mpigi and Bugiri in rural Uganda. Eighteen selected focus group discussions (FGDs), each internally homogeneous, and eight in-depth interviews (IDIs) were conducted among men and women. Data were collected on sociocultural beliefs and practices, barriers to modern contraceptive use and perceptions of and attitudes to contraceptive use. All interviews were tape recoded, translated and transcribed verbatim. All the transcripts were coded, prearranged into categories and later analyzed using a latent content analysis approach, with support of ATLAS.ti qualitative software. Suitable quotations were used to provide in-depth explanations of the findings.

**Results:**

Three themes central in hindering the uptake of modern contraceptives emerged: (i) persistence of socio-cultural beliefs and practices promoting births (such as polygamy, extending family lineage, replacement of the dead, gender-based violence, power relations and twin myths). (ii) Continued reliance on traditional family planning practices and (iii) misconceptions and fears about modern contraception.

**Conclusion:**

Sociocultural expectations and values attached to marriage, women and child bearing remain an impediment to using family planning methods. The study suggests a need to eradicate the cultural beliefs and practices that hinder people from using contraceptives, as well as a need to scale-up family planning services and sensitization at the grassroots.

## Introduction

Investing in family planning has been part of the global a global agenda in recent years with a focus on meeting Millennium Development goals four and five [1–3]. High birth rates not only affect maternal and child mortality but frustrates governments in the provision of social and health services to communities. Studies have shown great benefits of investing in family planning including reduced poverty levels, improvement in maternal and child survival, and women’s participation in the labor market [3–5]. However, over 200 million women in developing countries have an unmet need (proportion of married women in need for contraception to space or limit births but they are not using anything) for family planning even when there is a global call for promotion of and investment into family planning [6, 7]. Although global fertility estimates have been reducing over the years, and many countries have reached replacement fertility or lower, Sub-Saharan Africa as a whole still has a high total fertility rate (the highest of all the global regions) of 4.8 children born per woman compared to 1.7 births in developed countries [8, 9]. With a persistently high fertility rate of 6.2 children born per woman in 2011, Uganda has one of the fastest growing populations in the Sub Saharan region at a rate of 3.2% per annum [10]. This poses a great threat to the development and wellbeing of the Ugandan population as reflected in high under- five and maternal mortality rates. According to the 2011 Uganda Demographic and Health Survey estimates, there were 438 deaths of women per 100,000 live births and an infant mortality rate of 54 deaths per 1,000 live births. Despite government efforts to reduce high fertility levels and increase uptake of family planning services in Uganda, the contraceptive prevalence rate is only at 30 percent among married women which is the lowest in East Africa: the rates in Kenya Rwanda, and Tanzania are 45.5%, 36.4% and 34% respectively [11, 12].

Research elsewhere shows access barriers associated with health workers who only promote their preferred and available modern contraceptive methods [13, 14]. According to other studies, the explanation for unmet need and low contraceptive use is caused by fear of side effects, partners’ disapproval, limited method choice and knowledge and societal disapproval [5, 15–19]. Son preference has too been an issue in fertility especially in patrilineal societies, and this persists even with improvement in women’s education levels and socio-economic development [20]. In addition cultural barriers in in particular traditional preferences and desires for more children and lineage, have been highlighted as affecting the uptake of family planning [20, 21]. In the past decade Uganda introduced a population policy as a guiding tool in the implementation of population programs of which family planning is top of the agenda. However, the uptake and utilization of family planning services has continued to be extremely low [22]. This begs the question: Can the low utilization of family planning services in Uganda be attributed to the strong sociocultural settings and traditional beliefs that exist in patriarchal societies [23–25]. What is not known is whether, with improved education and socioeconomic development in Uganda, people’s cultural preference for more children and extending family lineage could have changed. This paper therefore investigates the influence of socio-cultural beliefs and practices that hinder the use of modern contraceptives in two rural Ugandan districts. We seek to examine what influences people to continue having more births even with the existence of effective modern methods of contraception.

## Methods

This was a qualitative study that was conducted using focus group discussions (FGDs) and in-depth interviews (IDIs) among community members. The design was deemed appropriate as it availed in-depth explanations of the prevailing practices regarding contraceptive use and cultural inhibitions in the study districts.

### Study setting

We collected data in the months of July and August 2012 from Bugiri and Mpigi districts of Uganda. These were rural districts selected from the eastern and central regions of Uganda. Though located in different regions of the country the two districts had a few similar characteristics including subsistence farming as the main economic activity, similar rainfall patterns, rainy seasons and populated by the same ethnic group of people called Bantu. The districts however belong to different cultural groups: Baganda for Mpigi and Basoga for Bugiri, with varying customs, languages, norms and values.

### Participants and data collection procedures

Pre-study visits were conducted in the sampled parishes with local leaders and proposed guides describing the study population and objectives. Focus group discussions (FGDs) were conducted in 18 different villages within the sampled sub-counties and parishes until saturation. These groups’ participants were selected based on homogeneity with reference to age, sex and residence. On the other hand, in-depth interviews were structured conversations with selected knowledgeable individuals within the sampled communities. Men and women who had the highest number of children ever born or fathered were legible to participate as in-depth interviewees. Discussions were carried out with female participants in the reproductive age categories of 15-24, 25-34 and 35-49, while males were aged 15-24, 25-34 and 35-54. One hundred fifty four (154) FGD participants were interviewed with an average of 8 people per FGD. Field guides were used in the identification of the participants and the location the sampled areas. Prior to data collection, the structured tools were adjusted following a pre-test for consistency and accuracy. Verbal consent was sought from all the participants after explaining to them the study objectives. Most of the procedure followed herein is suggested by qualitative researchers [26–28] Trained and experienced social science researchers using translated guides moderated the discussions while the note-takers captured detailed field notes in their notebooks. Audio recorders were also used to augment the field notes captured by the note-takers. Eight In-depth interviews were conducted with an equal distribution of selected females and males who had the highest number of children ever born and fathered in the communities respectively. The IDIs were selected to provide general perceptions about having many children born and the use of contraception. The languages used for the translated guides were in the local dialects of *Lusoga* and *Luganda* for Bugiri and Mpigi districts respectively were used. Discussions lasted 60 to 90 minutes. Socio-demographics characteristics of participants, including age, marital status, education, and number of children born were captured before the discussions. Participants were reimbursed for their transport expenses at the end of the discussions. Venues for the discussions were identified by one of the local council elders on the recommendation of the group participants. Transcribed data were later translated into English and audio recordings were used to cross-check the transcript as based on the steps recommended by Quin [28].

### Data analysis

Selected research assistants fluent in the local languages listened to audio tape recordings and transcribed verbatim data. The first and last author who established the coding scheme did multiple reading of the transcripts while making notes. Using latent content analysis the identified codes were prearranged into categories and later into emerging themes. The key issues related to cultural inhibitions towards contraceptive use and emerging themes were captured accordingly. Three major themes were identified First, persistence of sociocultural beliefs and practices promoting births like polygamy, extending lineage and replacement of the dead, gender based violence, power relations and twin myths. The second theme was continued reliance of traditional family planning practices and the third theme was misconceptions and fears about modern contraception. ATLAS.ti qualitative software was used in the organization and analysis of the data. Quotations are used in the presentation of the results.

### Ethical considerations

This study received ethical clearance from the Uganda National Council of Science and Technology (UNCST), which is the national approving body. Further approval was also sought from local leaders who had to be informed about the study before the data collection period. Prior to the group discussions a consent form was read out to the participants who were given a chance to ask questions and have a representative sign on their behalf. On consent the in-depth interviewees had a chance to retain copies of the signed consent forms in case of any other clarification and queries. To all the participants, issues of anonymity and confidentiality were emphasized. Assigned alias numbers were used to conceal focus group participants’ names and identifications.

## Results

Out of the eighteen focus groups discussions conducted, 8 were for males while 10 were for females. Table 1 presents more than half of the participants 60% were aged 20-39, 66% were married and 56% had primary education as the highest level attained. Two out of five participants 40% had 5 children and above, 35% were Catholics while 32% were Protestants. Eight in-depth interviews with an even distribution of males and females with an average of 8 children born were also conducted in the study districts. In total 162 people participated in the FGDs and IDIs. The results are discussed according to the into three emerging themes: perseverance of existing sociocultural beliefs and practices, continued reliance on traditional practices and contraception misconceptions and fears.

**Table 1:** Socio-demographic characteristics of participants

Characteristics	N=154	Percentage
**Age**		
19 & below	21	14
20-29	40	26
30-39	52	34
40-54	41	27
**Marital status**		
Single	32	21
Married	102	66
Divorced/separated/ widowed	20	13
**CEB**		
0	29	19
1-2	36	23
3-4	27	18
5+	62	40
**Religion**		
Catholic	54	35
Protestant/Pentecostal	50	32
Moslem	34	22
Other	16	10
**Education**		
None	19	12
Primary	86	56
Secondary	49	32
**No. FGDs**		
Males	8	44
Females	10	56

### Perseverance of socio-cultural beliefs and practices promoting births

The discussants’ views on cultural practices as hindrances to contraceptive use a contradictory. Participants claimed that while the traditional and cultural practices would not stop one from using modern contraceptives per se, there were situations which would compel one to refrain from not using modern methods. These situations included; extending family lineage, no or few boys already born, the most recent births being twins, competition between co-wives in a polygamous union, religious prohibition of contraception and replacement for the dead.

#### Extending family lineage and replacement of the dead

The belief and practice of extending family lineage is still held by most communities visited. Most of all male participants believed that women should produce many children to extend their lineage. A lot of value is attached to children, which implied that bigger families were most preferred. Bearing a son to be the heir to the husband was most revered by many women. Having an heir was extremely important for men too, so women without sons would continuously give birth to more children until a son is born. Meanwhile, to some female participants, births meant replacement of ancestors. This is demonstrated in the naming of children with reference to those who died. This implied continuity of clan’s existence and cultural values.

#### Twins myths

There is a belief that once a mother has had twin births they should not stop at twins. Both in-depth and female FGD participants reported this belief and attached a lot of value to it. It was believed to be abominable to have twins as the last-born children especially among participants from Mpigi district. There were many cultural practices and societal obligations for twin births in both districts including special names given to twins and to children or a child born after and before twin births. The parents too are given special names as a way of respecting them for having twins. Among the Basoga of Bugiri district, the father of the twins is referred to as “isabirye nantaloba” Similarly among the Baganda of Mpigi district have unique names given to parents of the twins. On one of the participants had this to say;

“For me what I hear people saying is that “Nalongo or Salongo” parents of twins do not stop giving birth at twins. There should be a follower to the twins called “KIIZA”, therefore if someone bears twins they have to get another child to follow the twins” (female FGD participant, 25-34 yrs, Mpigi).

Bearing twins comes with a lot of prestige particularly among the communities of Basoga and Baganda. There are special occasions that are organized by the clan leaders to appease the ancestors as well as name the children. There are special cultural dances and rhymes that are sung and to some extent people around indulge in provocative sex. The twin myth remains an impediment to contraceptive use as those who have had twin births try to have atleast one or more child, do not end with them, and through the mock twin dances and celebrations other women can easily conceive.

#### Polygamy

Half of all the focus group discussions maintained that the practice of polygamy, a man having more than one wife, was acceptable in most African traditional societies where it is deemed acceptable for a man to have more than one wife. With a lot of attachment to children and wealth, bearing many children would mean security for the mother. However polygamy was also considered to breed suspicion and competition among co-wives who struggle for societal approval and respect and this has pushed women into competing for their husbands’ love while producing many children. Therefore, women opt not use FP for fear that their co-wives might have more children than them. This was reported by most group discussions as demonstrated by one of the participants below:

“Most women in polygamous relationships are competing with their co-wives to have more children born. Competition comes in when the man is well off, has some money, domestic animals and land so when one produces few, her children would be cheated while sharing the fathers’ inheritance” (Male FGD participants, 35-54yrs, Bugiri).

#### Marital obligations regarding births

The societal expectation for married women is to give birth to many children. During the group discussions, participants mentioned that fertile women were most the preferred with prominence to those who produce throughout their reproductive ages. To many people marriage means births, therefore a married a woman is be expected to bear children frequently. When a woman does not bear children as frequently as the man wishes, she can easily be chased from her marital home. Besides, price bride is given with a hope that a woman would bear as many children as possible and that girls are cautioned accordingly.

“… in our society every married woman is expected to have as many children as possible. Those who cannot bear children are given undesirable names. Occasionally, some married women with few or no children are threatened by their partners to retrieve bride price from their families.” (Female in-depth participant, Bugiri).

Women are encouraged to be obedient and do as their husbands please. Therefore the perception held by many participants that men prefer having many children born is a push to continued high fertility. Continued births are a sign of respect and love for the husband, which is crucial in marriage.

#### Religious practices

Like any many other societies, there were beliefs attached to children related to the fact God was the source of life. Both male and female discussants mentioned that children are a gift from God and that all children come with a blessing. Ten group discussions mentioned that traditionally, every child is considered to come with special blessings and that it is God will provide for them. It does not matter how many children one has, God cares and provides for those children who are born. Relatedly, modern religions like Islam and Catholicism preach that all children are from God and that it is abominable to interfere with the will of God. According to these religions, the use of contraception is likened to the killing of innocent unborn children and only God should be responsible for limiting births. Therefore ardent followers would heed to the preaching of their elders.

“When it comes to religion, for example, the Muslims have to produce until the eggs are finished in the womb. However not only the Muslims, the Bible says go produce and fill the world. Muslims also have a belief that every child comes with his or her blessing” (Male In-depth participant Mpigi).

#### Gender violence and power relations

Some women have no autonomy in their homes as they continuously in have fears of domestic violence or being chased away for bearing few children. The community perception is that women have no say regarding the number of children to be born in a household. The notion that a man determines the number of children and is never limited his dominance was alluded to by a number of males and females in the discussions. A female in-depth participant had this to say:

“Men are difficult and always say they are the decision makers. For instance I had twins with my husband, however when I conceived again, they (health workers) called my husband to talk to him because I had complications. They (health workers) wanted me to stop giving birth but my husband did not want” (Female In-depth-Interview participant, Bugiri).

For some women the pressure from in-laws for young women to bear children is unbearable. Female participants said the interference by some in-laws was a hindrance in the use of modern contraceptive methods. They demand for many children to be borne by the daughters and sisters-in-law as that was the presumed purpose for marriage. This is illustrated below by one of the group discussions:

“…around here, there is a family where the son married and built near his mother’s home. The mother- in-law keeps telling her daughter-in-law that she has to produce till all her children get finished from the womb. She claims that if she had not produced her son then nobody would have helped her. Therefore such a woman would know that family planning is good but because the mother-in-law says she should produce all the children from her womb, the woman cannot do otherwise” (Female FGD participant, 25-34yrs, Bugiri).

### Existence of traditional family planning practices

The second major theme, which emerged from analysis, was perseverance of traditional practices for spacing and limiting births. In both districts, participants mentioned one or two traditional methods of preventing pregnancy and this was most common among both males and females aged 35 years and over. These included the use herbs, tying of traditional herbs around the waist, drinking of some concoctions mixed with water, using safe days/ withdrawal method, and tying the umbilical cord around the waist. In addition breast-feeding was mentioned by almost all the groups as a method commonly used for spacing births. When the herbs were used, these were administered by a herbalist or traditional doctor. A variety of different herbs were used, and their use differed too: some were drunk directly, others inserted in the woman’s vagina or tied around the waist, a practice locally known as use of “yirizi”.

“Yes, I heard that women usually go to traditional doctors who make them sit in herbs mixed with water for about an hour or so. Once these herbs enter inside the woman’s womb, she cannot conceive. The traditional doctor may give a woman a duration of 2 or 3 years when one would not conceive” (Male In-depth participant, Mpigi).

In some communities women use the umbilical cord that falls off their baby two weeks after birth. In such cases a herbalist mixes herbs with the baby’s umbilical cord which is then put in a waistband and tied around the breast-feeding mother.

“Yes; people here say that if a woman gets the child’s umbilical cord and mixes it with some traditional medicine then ties them together and keep them in a secret place or around the waist then this woman would not conceive until she unties the concoction in future” (Female In-depth participant Mpigi).

None of the participants claimed to know how the traditional waist bands affected fertility, they felt that all one had to do was to heed to the instructions given by the traditionalists as well as herbalists. If a woman wished to resume bearing children she would simply remove the waistband. There was also another view that a mixture of herbs with used sanitary pads would prevent a new mother from conceiving again. In some cases women were told to get some blood from the first menstrual cycle after delivery to be mixed with herbs which were placed in the house in a manner prescribed by the traditional healer. There were divergent views regarding the best location of the used sanitary pad or towel mixed with local herbs. Possibilities mentioned included burying it or hanging it in the kitchen, ceiling, at the doorway of the main house or in the master bedroom. The belief was that the one whose pads have been used would comfortably engage sex without fear for conception. One of the participants had this to say:

“Yes using my menstrual blood after delivery, I can tie it with herbs in a piece of paper and put it in the kitchen chimney where the smoke gets out. That method can easily prevent one from conceiving” (Females FGD participant, 35-49yrs, Bugiri).

Participants aged 35 years and above still held on to traditional beliefs and practices and had used them or knew someone who had used them. In almost all the female groups, participants were knowledgeable about the different traditional practices but this was not the case with the males.

“Not only here in Mpigi but in the whole of Uganda most people are still mainly using traditional/ cultural practices like consulting their ancestral spirits. There is no where you can go more than 3-4 steps without finding a traditional healer/ witch doctor in this community” (Male FGD participant, 35-54yrs, Mpigi ).

Furthermore there seemed to be knowledge gaps about the different traditional and cultural methods among the young adults (15-24) who said they were in a modern generation and would not know the old traditions and cultures.

“Those were methods uses a long time ago. Such traditional contraception methods of using herbs and cultural practices are no longer efficient. The elderly people who knew these herbs are no longer there as they died” (Male FGD participant 15-24yrs, Mpigi)

A few, however were less adamant, saying they had heard about the methods but had not come across people using them.

“We hear our elders were using traditional medicine, I do not know them. I doubt whether they still use them because every generation has its practices but what I know is that they give you herbs for drinking. Also there are women who cannot get pregnant when breastfeeding. A woman can spend 3 years breastfeeding her baby and when she chooses to stop, she can immediately conceive” (Female FGD participant 15-24yrs, Bugiri).

Elderly women who sometimes worked as Traditional Birth Attendants (TBAs) were widely reported to be knowledge custodians for cultural and traditional contraceptive practices. On several occasions, focus group participants said that if one needed advice one would consult elderly women. The number of children delivered by the elderly women did not seem to be an important indicator of their presumed knowledge; these elderly women were considered to be knowledgeable simply because they had lived so many years crossing different generations.

“Those were for a long time ago, the contraception methods of using herbs and cultural practices. The elderly people who knew these herbs are no longer there as they died” (Male FGD participant, 19-24yrs, Mpigi).

It did not matter whether one was related to the elderly woman or not as long as they had the herbs that one could use, that alone qualified them to be knowledgeable and full of experience. These guarded their assortments and could not share with their clients about the ingredients of the given herbs or medication.

“We have elderly women who are knowledgeable about the traditional methods that can be used to space births. These are respected in our communities for their expertise and advice. Many women in need of traditional methods do consult them” (Female FGD participant, 35-49yrs, Mpigi).

### Misconceptions and fears about modern contraceptives

The focus groups revealed that some people had resorted to using traditional and cultural practices because of the fear of perceived side-effects of modern contraceptives. Commonly held myths, fears and misconceptions were associated with prolonged bleeding, the birth of abnormal children and tumors in the womb. It was believed that those who used modern methods became infertile, as the methods were perceived to destroy ova, delay return to fertility, and caused cancer and bodily pains. Men feared that, women using modern contraceptives would undergo unhealthy weight gain or loss. This is illustrated by the following:

“If women use cultural practices or herbs or evoke spirits, with these they don’t get side effects because these can be untied and ones fertility restored. It won’t be like pills that will pile up in her stomach and bring harm. For instance traditional herbs like “mumbwa” luganda word meaning (clay) are easy for her to use since they are not harmful to her. Most women if they go in for cultural practices at the end of their reproductive period, they remain looking good but pills treat them badly. Sometimes you find them (women) losing or gaining unnecessary weight which is not the case with traditional methods” (Male FGD participant, 25-34yrs, Mpigi).

Hormonal methods were thought to be associated with side effects such as recurrent dizziness, lack of sexual libido and impromptu bleeding, and the fear of both these and infertility led women to opt for traditional practices instead of modern ones.

“… the modern methods have some side effects. People say that modern methods are bad, they have a belief that if you stop using the modern contraceptive methods and want a child, you will never get pregnant” (Male In-depth participant Mpigi).

#### Stigma and partner opposition

The fear of side effects has led to entrenched male opposition to modern contraception. This has spread from possibly justified fears such as of the prolonged bleeding which might accompany some forms of modern contraception for a few women, to fears of permanent infertility and adultery. This has led to stigmatisation of women who use modern family planning methods, who are given names in some communities like *“akongose”* in lusoga meaning being too slim. These are mocked for having few children born and are always referred to as prostitutes or adulterers.

#### Costs of modern contraception

There were perceived costs attached to use of modern methods of contraception. The use of traditional medicine was also attributed to lack of resources to buy modern contraceptive methods. They claimed that those women who could not afford to have modern contraceptives resorted to using the traditional or cultural practices. This did not mean that traditional medicine was given freely. However, the costs incurred in acquiring them were very low compared to those of modern family planning methods.

“…Because of the side effects of the modern methods, and also the fact that people are very poor and cannot afford the modern methods, they use the traditional ones which are free” (Male In-depth participant, Mpigi)

## Discussion

This qualitative study of socio-cultural inhibitors to the use of modern contraceptive techniques in rural Uganda shows that sociocultural expectations and values attached to marriage, women and children remain an impediment to using modern contraception methods. These are reflected in the societal norms, practices of polygyny, preference for large families, in the importance of extending lineage, twin myths, perceptions that men prefer many children and son preference. In addition, the attitudes of women as well as the society that they should produce until they have finished all the children in their womb, or until a desired number of children is reached is a key sociocultural inhibition to the utilization of contraception, which contributes to the persistence of high birth rate. These findings confirm the strong patrilineal connection and socio-cultural benefits that are accrued by families with many children born, observed by other researchers [21, 29, 30]. The impact of polygyny was emphasized by women: those who were not polygamous relationship felt the need to continuously give birth to prevent their husbands from wanting another wife, while those in such unions bore children in competition with their co-wives. Either way, the desire to please their husbands and acquire inheritance and security leads to high fertility [31]. Additionally, fear of domestic violence held by women indirectly pushes them to continue bearing more children as a means of being respectful. Gender based violence, particularly women being battered for reasons of barrenness, delaying to conceive, use of contraception without husbands approval and having few children, are serious impediments to the use of any method to limit or space births. These women leave in fear because they cannot go against the decisions of the household head least they suffer beatings [32, 33]. In patriarchal societies obedience of wives is highly regarded, therefore partner opposition to modern methods would mean women do not adher to health workers’ pleas to use them [34, 35]. It however demonstrates poor spousal communication especially with regard to birth spacing, limiting and desired number of children [36, 37].

The belief in children being God given was a pertinent issue that compelled people to have more births. Having children to replace the dead was a way of extending lineage. Given the high infant and child mortality in the country (54 and 90 per 1,000 live births respectively), many Ugandans still had fears regarding the small families, when not certain about the survival of the available children. This is rooted in the strong cultural settings and societal norms in most patriarchal communities, which are not only common in Uganda but in most communities that have strong preference for large families as evidenced [34, 35, 38]. Most of the participants were knowledgeable about the sociocultural and traditional practices used to limit and space births. The study findings however showed that young people aged 15-24 dissociated themselves from the traditional practices claiming that old-fashioned and elderly people used them. This implies that changing perceptions and practices make younger people appreciate the use of modern contraceptives and are more willing than older people to use these methods [19]. This finding is similar to results of other researchers that changing perceptions among young people have positively influenced their uptake of modern contraceptives in Uganda [39, 40]. Though these traditional practices still exist, some participants mentioned the futility of these methods especially in spacing births. Those who had used them reported failure of the traditional practices as most had conceived unknowingly. This probably explains the high unwanted and unplanned pregnancies reported in the recent demographic and health survey report [41, 42]. Furthermore, this result suggests an opportunity to increase the promotion and uptake of modern contraceptives which are more effective than the traditional ones in the reduction of high fertility levels [43].

Misconceptions regarding the modern contraceptives were still held by most participants who believed their use would cause infertility. These misconceptions disrupt consistent use or lead to discontinuation of modern contraceptive methods. The continued use of traditional and cultural methods could probably suggest a lack of appropriate and limited temporary modern methods with fewer perceived side effects. It is not surprising that some women simply end up conceiving for lack of appropriate method of contraception [44, 45]. The persistent use of cultural and traditional practices of limiting and spacing births was mainly attributed the fear of side effects, failure of modern contraceptives, costs of contraceptives and sociocultural obligations. Similar findings were reported from other studies [46, 47]. This study had some limitations. First, the findings generated herein may not be generalized to the whole of population given the diverse social, cultural and traditional beliefs and practices in the country. The discussions may not have provided the conclusive information regarding a particular tribe or culture given the heterogeneous community, migration and intermarriage within.

## Conclusion

This paper shows the persistence of traditional and sociocultural practices that frustrate existing reproductive health programs. The paper also demonstrates the need for increased investment in family planning sensitization campaigns, role-plays and training for both men and women in the rural communities in Uganda. Strategies toward such efforts would help increase the current contraceptive prevalence of modern contraception in the country.

### What is known about this topic

- Studies have shown great benefits of investing in family planning including reduced poverty levels, improvement in maternal and child survival, and women’s participation in the labor market;
- Knowledge of various contraception methods increases uptake of modern contraceptive use;
- Divergent views regarding social cultural practices and their perceived influence on use of contraception in high fertility countries.

### What this study adds

- This is one of few to examine gendered perceptions and inhibitions regarding women’s use of modern contraception and family planning services in a sub-Saharan African setting;
- The study presents indirect influence of socio cultural practices on the use of modern contraception in a high fertility country amidst universal knowledge of contraceptive methods;
- The study is based on rigorous data collection and analytical methods based on a study population of both males and females with age 15-60. The methodology used herein provides in depth understanding of the hindrances to use of contraception.
